# Robust Sampling of Defective Pathways in Alzheimer’s Disease. Implications in Drug Repositioning

**DOI:** 10.3390/ijms21103594

**Published:** 2020-05-19

**Authors:** Juan Luis Fernández-Martínez, Óscar Álvarez-Machancoses, Enrique J. deAndrés-Galiana, Guillermina Bea, Andrzej Kloczkowski

**Affiliations:** 1 Group of Inverse Problems, Optimization and Machine Learning, Department of Mathematics, University of Oviedo, C/Federico García Lorca, 18, 33007 Oviedo, Spain; UO217123@uniovi.es (Ó.Á.-M.); andresenrique@uniovi.es (E.J.d.-G.); guillermina.bea@icloud.com (G.B.); 2 DeepBioInsights, C/Federico García Lorca, 18, 33007 Oviedo, Spain; 3 Department of Informatics and Computer Science, University of Oviedo, C/Federico García Lorca, 18, 33007 Oviedo, Spain; 4 Battelle Center for Mathematical Medicine, Nationwide Children’s Hospital, Columbus, OH 43205, USA; Andrzej.Kloczkowski@nationwidechildrens.org; 5 Department of Pediatrics, The Ohio State University, Columbus, OH 43205, USA

**Keywords:** Alzheimer’s disease, mild cognitive impairment, pathway analysis, deep pathways sampling, drug repositioning

## Abstract

We present the analysis of the defective genetic pathways of the Late-Onset Alzheimer’s Disease (LOAD) compared to the Mild Cognitive Impairment (MCI) and Healthy Controls (HC) using different sampling methodologies. These algorithms sample the uncertainty space that is intrinsic to any kind of highly underdetermined phenotype prediction problem, by looking for the minimum-scale signatures (header genes) corresponding to different random holdouts. The biological pathways can be identified performing posterior analysis of these signatures established via cross-validation holdouts and plugging the set of most frequently sampled genes into different ontological platforms. That way, the effect of helper genes, whose presence might be due to the high degree of under determinacy of these experiments and data noise, is reduced. Our results suggest that common pathways for Alzheimer’s disease and MCI are mainly related to viral mRNA translation, influenza viral RNA transcription and replication, gene expression, mitochondrial translation, and metabolism, with these results being highly consistent regardless of the comparative methods. The cross-validated predictive accuracies achieved for the LOAD and MCI discriminations were 84% and 81.5%, respectively. The difference between LOAD and MCI could not be clearly established (74% accuracy). The most discriminatory genes of the LOAD-MCI discrimination are associated with proteasome mediated degradation and G-protein signaling. Based on these findings we have also performed drug repositioning using Dr. Insight package, proposing the following different typologies of drugs: isoquinoline alkaloids, antitumor antibiotics, phosphoinositide 3-kinase PI3K, autophagy inhibitors, antagonists of the muscarinic acetylcholine receptor and histone deacetylase inhibitors. We believe that the potential clinical relevance of these findings should be further investigated and confirmed with other independent studies.

## 1. Introduction

Alzheimer’s disease (AD) is the most common cause of dementia associated with aging, causing the loss of intellectual and social skills. The causes of Alzheimer’s are not yet fully understood. The Early-Onset form of Alzheimer’s Disease (EOAD) is due to mutations in chromosome 21, causing the formation of abnormal amyloid protein (APP) [1], and mutations on chromosomes 14 and 1 leading to abnormal presenilins 1 and 2 which undergo cleavage of APP [2,3]. However, the causes of Late-Onset Alzheimer’s Disease (LOAD) are not yet completely understood. The prevalent common view is that they likely include a combination of genetic, environmental, and lifestyle factors that determine the risk for developing the disease. 

The main milestones in the genetic analysis of LOAD go from the early descriptions of *apolipoprotein E* (*APOE*) gene polymorphisms in LOAD and *APP* gene mutations in EOAD in 1991 to the *presenilin 1* (*PS1*) and *presenilin 2* (*PS2*) gene mutations in EOAD in 1995. Since 2009 until now new polymorphisms influencing the risk of LOAD are being reported every year. Ricciarelli et al. (2004) investigated gene expression in Alzheimer’s disease and aging, reporting 314 genes that were differentially expressed in LOAD cerebral cortex, and confirming via reverse transcription polymerase chain reaction (RT-PCR) the increased expression of the interferon-induced protein 3 in LOAD brains [4]. Kong et al. (2009) performed independent component analysis (ICA) of Alzheimer’s DNA microarray gene expression data [5]. They identified more than 50 significant genes with high expression levels in severe LOAD, representing immunity-related proteins, metal binding proteins, membrane proteins, lipoproteins, neuropeptides, cytoskeleton proteins, cellular binding proteins, and ribosomal proteins. Our understanding of the etiology and pathogenesis of LOAD was predominantly influenced by recent developments of genetics [6]. Particularly, genome-wide association studies (GWAS) identified over 20 genetic loci associated with LOAD. 

Recent genetic data continue to support the amyloid hypothesis of LOAD with protective variants being found in the amyloid gene, and both common low-risk and rare high-risk variants being discovered in genes that are part of the amyloid response pathways. These data support the view that genetic variability in how the brain responds to amyloid deposition is a potential therapeutic target for the disease, and are consistent with the notion that anti-amyloid therapies should be initiated early in the disease process (Hardy et al. 2014) [7]. Genome-wide association studies involved genes related to three main ontological pathways: [1] Endosomal vesicle recycling (phosphatidylinositol binding clathrin assembly protein (*PICALM*) coding gene, bridging integrator-1 protein (*BIN1*) coding gene). (2) Cholesterol and lipid metabolism (apolipoprotein E (*APOE*) gene, clusterin protein (*CLU*) coding gene, and binding cassette subfamily A member 7 protein (*ABCA7*) coding gene) [3]. Innate immune system (Clusterin complement C3b/C4b receptor 1 (*CR1*) gene, membrane-spanning 4-domains subfamily A (*MS4A*) gene cluster, triggering receptor expressed on myeloid cells 2 (*TREM2*) gene) [8]. 

Giri et al. (2016) overviewed the genes associated with LOAD, noting that the majority of genes associated with LOAD cluster roughly within three main pathways: lipid metabolism, inflammatory response and endocytosis [9]. Additionally, several signaling pathways associated with LOAD might modulate various processes, such as the reduction of amyloid-β aggregation and inflammation, regulation of mitochondrial dynamics, and increased neuronal activity [10]. 

The difficulty to define some common mechanisms that could be responsible for development of LOAD is partly due to the high degree of underdeterminacy that is present in all genetic experiments. Besides, no single gene can describe complex diseases such as Alzheimer’s, caused by a combination of genetic, environmental, and lifestyle factors. Complex diseases do not obey the standard Mendelian patterns of inheritance, and it is well known that genes involved in complex diseases work in synergy (see for instance [11]). 

Pathway and network analysis is a good alternative to understand *Omics* data, and allows finding distinct cellular processes and signaling pathways that are associated with the set of differentially expressed genes. Pathway analysis needs databases with pathway collections and interaction networks, and programming packages to analyze the data. The most popular freely available public collections of pathways and interaction networks are Kyoto Encyclopedia of Genes and Genomes (KEGG) [12] and REACTOME [13]. Pathway and network analysis of cancer genomes is currently used for better understanding of various types of tumors [14]. Dimitrakopoulos and Beerenwinkel (2017) reviewed several computational methods of the identification of cancer genes and the analysis of pathways [15]. For AD, Mizuno et al. (2012) developed a publicly available pathway map called AlzPathway (http://alzpathway.org/) that comprehensively catalogs signaling pathways in AD using CellDesigner [16]. AlzPathway is currently composed of 1347 molecules and 1070 reactions in neuron, brain blood barrier, presynaptic, postsynaptic, astrocyte, and microglial cells and their cellular localizations. There are still some outstanding challenges concerning both annotations and methodologies [17]. The annotation challenges are due to low-resolution of available databases; while the methodological challenges concern mainly finding the set of genes that are indeed related to the disease and understanding the dynamical nature of biological systems and the effect of external stimuli. 

In this paper, we try to address the first methodological challenge related to the phenotype prediction problem, i.e. the development of robust computational methods of linking the cause (genotype) and the effect (phenotype). Researchers typically use sets of differentially expressed genes, but fold change is sensible to the presence of noise in genetic data and in the wrong class assignment of the samples [18]. 

The holdout sampler [19] looks for different equivalent high discriminatory genetic networks that are related to the uncertainty space of the classifier that is used to predict the phenotype. The holdout sampler generates different random 75/25 data bags (or holdouts): 75% of the data in each bag is used for learning and 25% for blind validation. For each of these bags the small-scale genetic signatures (header genes) are determined. The posterior analysis consists of finding the most frequently sampled genes taking into account all the highly predictive networks, that is, the small-scale genetic signatures with high validation accuracy. The biological pathways can be identified performing posterior analysis of these signatures established during the cross-validation holdouts and plugging the set of most frequently sampled genes into ontological platforms. That way, the effect of helper genes whose presence might be due to noise or to the high degree of underdeterminacy of these experiments is damped. As we briefly explain in the next section, this algorithm is inspired by the sampling of the equivalence region of a regression problem using bootstrapping (random data sampling with replacement) to find different sets of equivalent predicting parameters. 

We show the application of this algorithm to the analysis of the genetic pathways involved in LOAD and mild cognitive impairment (MCI), obtaining an unexpected association with influenza viral RNA transcription and replication as the main mechanisms in LOAD and MCI development. Neurodegenerative diseases could be induced by chronic and viral infections that may lead to a loss of neural tissue in the central nervous system. It has published rare instances in which acute severe encephalitic viral diseases directly cause transient symptomatic Parkinson Disease [20]. Besides, in the comparison of the LOAD patients vs. healthy controls (HC) we have also compared the altered genetic pathways derived by using various sampling algorithms to probe the hypothesis of biological invariance [21], that is, the genetic pathways that are involved in the disease development should be independent of numerical algorithm (classifier and sampling algorithm) that is used to unravel them. The discrimination between LOAD and MCI could not be clearly achieved and their joint (LOAD + MCI) difference from healthy controls can be achieved with a common list of genes found in their individual comparisons (LOAD vs. HC and MCI vs. HC). This fact is somehow expected as MCI may represent an early stage of LOAD and the genetic mechanism may be the same. 

Finally, based on the results of these analyses we show some implications for drug repositioning for LOAD-MCI.

## 2. Results 

### 2.1. Comparison of Late-Onset Alzheimer’s Disease (LOAD) Patients and Healthy Controls

Table 1 shows the list of most discriminatory genes of the phenotypes of the LOAD patients compared with HC. In this case the predictive accuracy estimation is based on Leave-One-Out-Cross Validation (LOOCV) by averaging the LOOCV predictive accuracy over all samples of the validation dataset in each bag and involves a simple k-Nearest-Neighbor classifier in the reduced set of high discriminatory genes (small-scale signature). 

Table 2 shows the most frequently sampled genes (sampling frequency higher than 0.7%) detected by the holdout algorithm. We also provide the mean of the expression in each group, the fold change, the Fisher’s ratio, and the sampling frequency. Table 3 shows the same analysis by using the Fisher’s ratio sampler (with a sampling frequency higher than 0.35) and Table 4 shows the results obtained with Random Forest (with a sampling frequency higher than 0.19). The sampling frequencies are varying and depend on the sampling algorithm.

**Figure 1:**
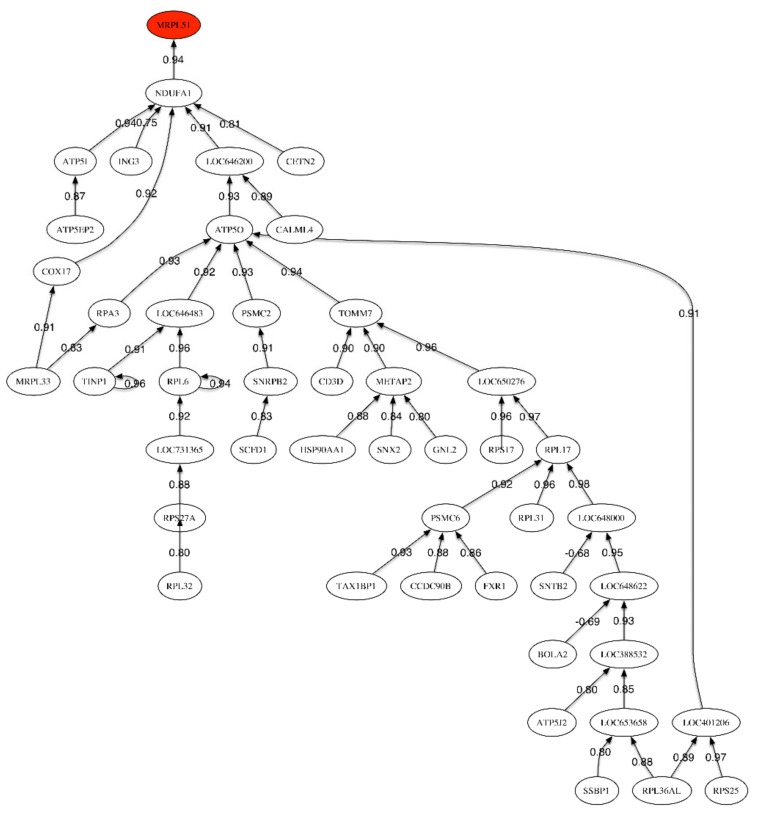
Correlation network for the LOAD vs. Healthy Control phenotype.

Table 5 shows the pathway analysis using the list of most highly sampled genes together with the summary of the results obtained for all the comparisons performed in this paper. The scores relative to these pathways are given in Table S1 as supplementary material. We have used the sampled genes with a frequency higher than 0.2, since this set contains enough discriminatory genes to perform the pathway enrichment. This sampling frequency has been judged to be optimum in the enrichment analysis. Figure 1 shows the correlation network between the most discriminatory genes of the LOAD vs. healthy control phenotype and serves to explain how the most discriminatory genes are interrelated and control gene expression.

### 2.2. Comparison of Mild Cognitive Impairment (MCI) Patients and Healthy Controls

**Figure 2:**
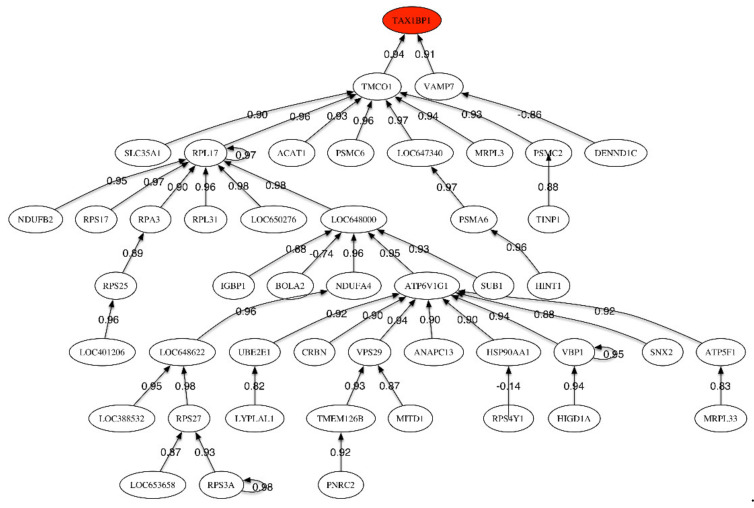
Correlation network for MCI vs. Healthy Control phenotype.

Table 6 shows the list of most discriminatory genes for the MCI vs. control phenotype discrimination. Table 7 shows the most discriminatory genes sampled in different networks for the Control vs. MCI phenotype found. In this case we have considered a sampling frequency higher than 0.38%. Table S2 (given in Supplementary Materials) shows the scores relative to the main genetic pathways involved in MCI vs. HC. Figure 2 shows the correlation network between the most discriminatory genes of the MCI phenotype. 

### 2.3. Comparison of MCI and Alzheimer’s Disease (AD) Patients

**Figure 3:**
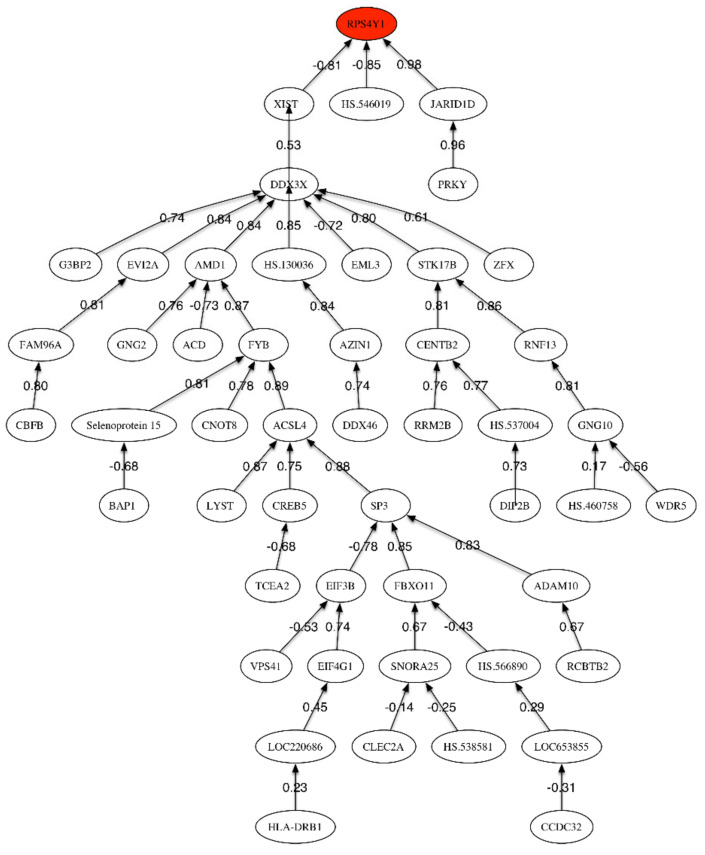
Correlation network for MCI vs. LOAD phenotypes.

Table 8 shows the list of most discriminatory genes for the MCI vs. LOAD phenotype discrimination. Table 9 shows the most discriminatory genes sampled in different networks for the LOAD vs. MCI phenotype with a sampling frequency higher than 0.5%. Table S3 (given in Supplementary Materials) shows the scores relative to the main genetic pathways in the discrimination between MCI and LOAD. Figure 3 shows the correlation network between the most discriminatory genes of the LOAD/MCI phenotype.

### 2.4. Comparison of MCI+LOAD Patients with Healthy Controls

Table 10 shows the list of most discriminatory genes for the MCI vs. LOAD phenotype discrimination. Table 11 shows the most discriminatory genes sampled in different networks for the LOAD + MCI vs. HC phenotype with a sampling frequency higher than 0.5%. Table S4 (given in Supplementary Materials) shows the scores relative to the main genetic pathways involved in the MCI + LOAD vs. HC discrimination. This last classification has been performed since the difference between MCI and LOAD cannot be easily established.

Table 12 summarizes the most important findings found in the main comparisons.

## 3. Discussion

### 3.1. LOAD vs. Healthy Controls Classification

In the LOAD vs. HC comparison, the maximum Fisher’s ratio obtained was 1.29 and corresponds to *MRPL51*. Only six genes have a Fisher’s ratio higher than 1. This fact gives an idea about the discriminatory power of gene expression data. The highest accuracy (79.52%) was obtained just with the two first genes: *MRPL51* and *CETN*. This accuracy was increased up to 84% by using a genetic signature of 21 genes that have been sampled within the set of most discriminatory genes. This signature is shown in Table 12, that serves to summarize the results. The most frequently sampled genes were *RPL36AL*, *MRPL51*, *LOC4011206*, and *RPS27A* (Table 2). These five genes are underexpressed in LOAD. 

*RPL36AL* is a protein-coding gene involved in metabolism and viral mRNA translation. It has been found that the overexpression of this gene is associated with cellular proliferation in hepatocellular carcinoma [22]. *MPRL51* is also a protein-coding gene related to mitochondrial translation. Recently it has been found that mitochondrial genes are altered early in blood in LOAD and MCI, showing reduced expression of *OXPHOS* (oxidative phosphorylation) genes. *RPS27A* is also a protein-coding gene involved in interferon gamma signaling pathway and activated *TLR4* signaling involved in immune responses. *CETN* (Centrin-1) is a protein-coding gene that plays a fundamental role in microtubule organization. *LOC4011206* is a non-characterized gene.

The main pathways with high scoring matches found in the LOAD vs. HC discrimination are viral mRNA translation, influenza viral RNA transcription and replication, gene expression, mitochondrial translation, rRNA processing, and metabolism (Table S1). Other pathways involved are organelle biogenesis, HIV life cycle, antigen presentation, and TCR signaling. Besides, Table 5 shows the comparison of these pathways to those found by the Fisher’s ratio sampler and the Random Forest sampler. It can be observed that all the samplers depicted similar pathways. Besides the most important biological processes involved (see Table 12) also pointed in the same direction. 

The correlation network (Figure 1) shows one unique link relating the header gene *MRPL51* to *NDUFA1*, which is involved in the mitochondrial respiratory chain pathway for the generation of cellular energy. The correlation between both genes is very high and positive (0.94). The main sub-tree develops under *LOC646200* (RPL22-60S ribosomal protein L22-heparin binding protein HBp15). This protein can bind specifically to Epstein–Barr virus-encoded small RNAs.

### 3.2. MCI vs. Healthy Controls Classification

In the MCI vs. HC comparison, the maximum Fisher’s ratio was 1.47, and corresponds to *TAX1BP1*. The highest accuracy (77.17%) was obtained with the first 59 most discriminatory genes. Table 6 shows only genes of this signature with Fisher’s ratio greater than 1. This accuracy was increased up to 81.5% by using a genetic signature of 13 genes (given in the summary Table 12) that have been sampled within the set of most discriminatory genes. 

The most important sampled genes (Table 4) are underexpressed in MCI samples with respect to healthy controls. Only *DENND1C*, *SULT1A3* are overexpressed in MCI. *DENND1C* is a protein-coding gene involved in clathrin-mediated endocytosis that seems to play an important role in AD [23]. *SULT1A3* is a protein-coding gene related to sulfotransferase enzymes that catalyze the sulfate conjugation of many hormones and neurotransmitters. It has been found that Alzheimer’s subjects have a significant lower *SULTA13* copy number compared to healthy controls [24]. Induction of *SULTA13* significantly protects cells from dopamine neurotoxicity. The effect of dopamine in AD has been discussed in the literature [25]. The number of most-frequently sampled genes with respect to the LOAD vs. HC discrimination has increased, but the sampling frequencies of these genes have decreased. This result could be interpreted in the sense that the MCI discrimination vs. HC is more ambiguous than the LOAD vs. HC discrimination, and might correspond to the fact that MCI is an earlier stage than LOAD. 

The pathways with higher scores found by the Holdout sampler are given in Table S2 and involve similar pathways to the LOAD vs. HC comparison (viral mRNA translation, gene expression, influenza viral RNA transcription and replication, metabolism of proteins, rRNA processing in the nucleus and cytosol, ubiquitin-proteasome proteolysis, antigen processing, cell cycle checkpoints, metabolism, HIV life cycle, mitotic metaphase and anaphase, *CLEC7A* (Dectin1 signaling), cellular senescence, TCR signaling, etc.). The main biological processes involved are also similar to the LOAD vs. HC comparison. Although not shown in the paper, the other samplers also depicted similar pathways.

The header gene in the correlation network (Figure 2), *TAX1BP1*, is positively correlated to *TMC01* and *VAMP7*, which is only correlated to *DENND1*. *TAX1BP1* encodes a HTLV-1 tax1 binding protein that interacts with *TNFAIP3* (tumor necrosis factor (TNF) alpha induced protein 3) and inhibits TNF-induced apoptosis. Among its related pathways are apoptosis, autophagy, and innate immune system. The degradation of this protein by caspase-3-like family proteins is associated with apoptosis induced by TNF. This protein might also have a role in the inhibition of inflammatory signaling pathways. Interestingly, the caspases have been also found to be involved in ALS. 

The most important sub-tree in the correlation network concerns *TMC01*, that plays a key role in calcium homeostasis. Dysregulation of calcium homeostasis in Alzheimer’s disease has been pointed by Kawahara (2004), Small (2009), Brawek and Garaschuk (2014) [26,27,28]. *TMCO1* is positively correlated to *RPL17* (Ribosomal Protein L17). Among its related pathways are viral mRNA translation and MAPK signaling. *VAMP7* is a protein-coding gene involved in the targeting and/or fusion of transport vesicles to their target membrane during transport of proteins. This gene has multiple interesting functions according to the Human Gene Database.

### 3.3. MCI vs. LOAD Classification

MCI represents an early stage of AD, and therefore the genetic background of LOAD might be shared by an important subgroup of MCI subjects. However, not all MCI subjects convert to LOAD since they may also convert to other diseases or remain stable over time. Therefore, this comparison should be interpreted as a temporal difference between both conditions, and the analysis was done even though it was not expected to find highly discriminatory genes. 

The highest accuracy (68%) was obtained with the first 118 genes, showing that, as expected, the difference between LOAD and MCI could not be clearly discriminated. Besides, to support this fact, only the three first genes in Table 5 have a Fisher’s ratio greater than 0.5: *RPS4Y1*, *HLA-DRB1*, and *JARID1D*. This accuracy was increased up to 74% by using a genetic signature of 8 genes that have been sampled within the set of most discriminatory genes: *RPS4Y1*, *FAM96A*, *HS.460758*, *CLEC2A*, *SNORA25*, *SMCHD1*, *GIMAP2*, *MAP2K2*. Some of these genes are related to the *RANK* (Receptor activator of nuclear factor-kappa B)-signaling pathway. The most important pathway involves the regulation of apoptosis by protein ubiquitination and degradation, which is one of the major mechanisms to regulate apoptotic cell death (Yang and Yu, 2003) [29]. A regulated balance between cell survival and apoptosis is essential for normal development and homeostasis of multicellular organisms. Defects in control of this balance may contribute to autoimmune disease, neurodegeneration, and cancer. A second important pathway concerns regulation of stress-activated MAP kinases by G protein signaling, that has an important role in the immune system [30] and has been a target in LOAD [31]. Besides, it is interesting to observe that within the set of most frequently sampled genes shown in Table 9 some genes are underexpressed and other overexpressed. This is different than in previous comparisons (MCI and LOAD vs. HC) where most of the discriminatory genes were underexpressed. The main pathway related to the underexpressed genes is chromatin regulation/acetylation. The epigenetic alterations in AD have been outlined by Sanchez-Mutt and Gräff (2015) [32]. The overexpressed genes are related to the regulation of activated PAK-2p34 by proteasome mediated degradation as main pathway. In both cases, pathways related to the immune system response are involved. The correlation network (Figure 3) has one main sub-tree relating *RPS4Y1* and *XIST*, and two other final nodes (*JARID1D* and *HS.546019*). Xist (X-inactive specific transcript) is an RNA gene on the X chromosome of the placental mammals that acts as a major effect of the X inactivation process. The X-chromosome instability phenotype in LOAD has been studied by Bajić et al. (2009) [33]. Additionally, Barati and Mansour (2015) [34] pointed that Xist has the highest level of overexpression in LOAD using microarrays expression techniques.

### 3.4. MCI+LOAD vs. Healthy Controls Classification

The maximum Fisher’s ratio (1.22) in this comparison (Table 8) corresponds to *LOC401206* (*RPS25P6*) that according to Aceview (NCBI) is an intronless pseudogene derived from the *RPS25* gene. *RPS25* (Ribosomal Protein S25) is a protein-coding gene. Among its related pathways are viral mRNA translation and activation of the mRNA. Within the set of most discriminatory genes we found *MRPL1* and *TAX1BP1* that also appeared in the LOAD and MCI vs. HC individual comparisons. Only *SULT1A3* is overexpressed in MCI and LOAD in the set of high discriminatory genes with FR greater than 0.8. The major pathways (shown in Table S4 provided as supplementary material) and biological processes involved are similar to those of the LOAD and MCI vs. HC comparisons, reinforcing the viral hypothesis found in previous comparisons. 

### 3.5. Implications in Drug Repositioning

Regarding therapeutics GeneAnalytics identified bortezomib and carfilzomib as potential compounds acting on some of the genes that are involved in the deregulated LOAD and MCI pathways. Bortezomib and carfilzomib are proteasome inhibitors used for multiple myeloma and mantle cell lymphoma. Nevertheless, GeneAnalytics does not provide information if these compounds act in the right direction to regulate genes involved in LOAD and MCI. Therefore, this finding indicates only the genetic processes and the genes that are involved. 

A more detailed drug repositioning was performed via Dr Insights (Chan et al., 2019) that uses the Connectivity Map (CMAP) transcriptomic experiments in different types of cell lines [35,36]. 

Table 13 shows the results of drug repositioning via Dr. Insights, using the list of most discriminatory genes identified by the holdout sampler. This analysis highlighted the importance of different compounds analyzed in breast cancer cell lines (MCF7) and one compound in prostate cancer cell line (PC3). In this table for each drug we also show the pathways that are affected by the genes whose expressions are increased or decreased as deduced from CMAP.

Emetine and its desmethyl analog cephaeline are isoquinoline alkaloids, which is a large class of nitrogen-rich natural compounds. Isoquinoline alkaloids are not a structurally homogeneous group, and their properties depend on the different degrees of oxygenation and intramolecular arrangements. Emetine is used for the treatment of amebiasis and is a component of ipecac syrup. Emetine is both myotoxic and cardiotoxic. Some isoquinoline alkaloids, such as galantamine, have been approved to treat MCI and other memory impairments. Its mechanism of action consists in cholinesterase inhibition that prevents the breakdown of the neurotransmitter acetylcholine, increasing its level in the synaptic cleft and in brain areas lacking cholinergic neurons. This treatment does not cure the condition but slows the rate of cognitive decline. Recently, different isoquinoline alkaloids have been studied as potential compounds for the treatment of LOAD [37,38]. 

This drug affects the tumor necrosis factor (TNF)-related apoptosis-inducing ligand (TRAIL) which has been shown to be unregulated in HIV-1-infected and immune-activated macrophages. TRAIL is also induced on neuron by beta-amyloid protein, an important pathogen for Alzheimer’s disease [39]. 

It has been shown that neutralization of *TNFSF10* ameliorates functional outcome in a murine model of Alzheimer’s disease [40,41]. Besides, it is evidenced that necroptosis is a major driver on neuron cell death in neurodegenerative diseases [42]. Additionally, among the pathways associated with genes whose expression has been decreased, the most important are related to cellular senescence and cellular response to stress.

Tanespimycin is antitumor antibiotic used for the treatment of leukemia and different types of solid cancer, whose mechanism of action consists of inhibiting Hsp90 (heat shock protein 90), which is a chaperone protein that assists other proteins to fold properly, stabilizes proteins against heat stress, and aids in protein degradation. Oxidative stress may cause Hsp60 structure modifications leading to loss of Hsp60 functions with the consequences of protein misfolding, aggregation and deposition. Besides, Hsp90 downregulation may induce the reduction of Tau hyperphosphorylation and aggregation and may trigger the so-called stress response. That is, in the presence of cellular stress and Hsp90 inhibitors, Heat Shock Factor 1 (HSF-1) protein dissociates from the chaperone, reaches the nucleus, inducing the activation of heat shock genes and of the stress response via the production of Hsp90, Hsp70, and Hsp40, restoring protein homeostasis [43]. 

The pathways associated with the genes whose expression has been increased are linked to the regulation of HSF1 heat shock response and unfolded protein response. The genes with decreased expression control apoptosis and signaling by TGF-beta receptor complex through a phosphorylated receptor SMAD (R-SMAD).

Wortmannin is a potent PI3K inhibitor, that serve to inhibit different phosphoinositide 3-kinase enzymes, which are part of the PI3K/AKT/mTOR pathway, an important signaling pathway for many cellular functions such as growth control, metabolism and translation initiation. Wortmannin and LY-294002 are autophagy inhibitors [44]. The main pathways related to the genes with increased expression are: toxicity of botulinum toxin type D, Insulin Receptor Signaling (IRS) activation, estrogen biosynthesis, collagen biosynthesis and growth hormone receptor signaling. Botulinum toxin is a neurotoxic protein produced by the *Clostridium botulinum* that prevents the release of the neurotransmitter acetylcholine from axon endings. 

The main pathways related to the genes whose expression are decreased are related to RNA modification in the nucleus, regulation of TP53 activity through methylation, fatty acyl-CoA biosynthesis, and galactose catabolism.

Biperiden is a muscarinic antagonist that blocks the activity of the muscarinic acetylcholine receptor, and has effects in the central and peripheral nervous systems. It has been used in the treatment of Parkinsonism [45]. The increased expression genes pathways are related to the Nuclear Pore Complex (NPC), which is the largest protein complex in the cell and also to the HIV life cycle; while the genes whose expression has been decreased are related to the secretin family receptors, that are involved in numerous key neurotransmitter systems in the brain and seem to be disrupted in Alzheimer’s disease (AD) hemostasis, and also in the regulation of the adaptive immune system [46].

Trichostatin A is a histone deacetylase inhibitor (HDACi). Acetylated histones and DNA methylation play important role in Multiple Sclerosis (MS) [47,48]. The overexpressed genes are related to glutamate neurotransmitter release cycle, antigen activation of B-cell receptor and Collapsin Response Mediator Proteins (CRMPs) in Sema3A signaling that modulate the immune system in neurological disorders with inflammatory components [49]. Glutamate neurotransmission is critical for synaptic plasticity and survival of neurons. However, excessive activity causes excitotoxicity and promotes cell death. This constitutes a potential mechanism of neurodegeneration occurred in Alzheimer’s disease [50]. The glutamate pathway has been found to be crucial in the development of Chronic Fatigue Syndrome (CFS) in cancer patients treated with radiotherapy [51]. 

The decreased expression genes are related to SMAD transcription factors, which play the key in the most versatile cytokine signaling pathways via the Transforming Growth Factor-β (TGFβ) pathway. The role of these factors in AD has been investigated [52]. Cyclin D, synthesized in G1 phase, is involved in regulating cell cycle progression, and is activated through phosphorylation. Overexpression of cell cycle proteins of peripheral lymphocytes (CDK2, CDK4, CDK6, cyclin B, and cyclin D) was observed in AD patients [53]. Finally, the last pathway concerns the transcriptional activation of mitochondrial biogenesis that controls the energy generating functions of mitochondria in accordance with the metabolic demands. The last drug that has been repositioned with high score is LY-294002, which is a PI3K inhibitor, like wortmannin. The main pathways are associated with RNA polymerase promoter, and adaptive immune system response, among others.

Other drugs that appear to be involved with lower scores are: saquinavir-MCF7 (5.2 × 10^−6^), sirolimus-MCF7 (1 × 10^−7^), and valproic acid-PC3 (5 × 10^−5^, 2 × 10^−4^). Saquinavir is an antiretroviral drug used to treat or prevent HIV/AIDS. Saquinavir is a protease inhibitor that cleaves protein molecules into smaller fragments. Sirolimus (also known as rapamycin) is an immunosuppressant, that inhibits activation of T-cells and B-cells by reducing their sensitivity to interleukin-2 (IL-2) through mTOR inhibition. Finally, valproic acid is a medication primarily used to treat epilepsy and bipolar disorder. Proposed mechanisms for the valproic acid include increasing brain levels of gamma-aminobutyric acid (GABA), blocking of voltage-gated sodium channels, and inhibiting histone deacetylases.

## 4. Materials and Methods

We performed the pathway analysis of a cohort of patients with Late Onset Alzheimer Disease (LOAD), mild cognitive impairment (MCI), and healthy subjects (controls) (HC). The source of data were Alzheimer case-control samples originated from the EU funded AddNeuroMed Cohort, which is a large cross-European AD biomarker study relying on human blood as the source of RNA (Sood et al., 2015) [54]. The dataset contains 38323 probes and 329 samples (145 LOAD, 80 MCI, and 104 healthy controls). These authors identified a set of 150 probe sets that could be used in a diagnosis of Alzheimer disease (AD) based on gene expression. This multi-tissue RNA signature, extracted from peripheral blood samples, could be used as a diagnostic tool. Nevertheless, its predictive value has been recently discussed and received some criticism [55,56]. 

We performed a retrospective cohort study using a novel machine learning and uncertainty methodology to sample different combinations of highly predictive genes for four different phenotype prediction problems, identifying the discriminatory genetic pathways in each case: LOAD vs. HC; MCI vs. HC, MCI vs. LOAD and MCI + LOAD vs. HC. Identification of the deregulated (or defective) genetic pathways is critical to establish personalized therapies. The potential relevance of these findings could be further investigated in clinical studies.

### 4.1. Sampling Defective Pathways in Phenotype Prediction Problems

To understand the uncertainty in phenotype prediction problems and the need of robust methods of sampling, we first present the existing inherent uncertainty in a simple linear regression. 

The least squares fitting of a linear model $y^{*}\left(x\right)=a_{0}+a_{1}x$ to a set of experimental data $\left\{\left(x_{1},y_{1}\right),\;\left(x_{2},y_{2}\right),\ldots ,\;\left(x_{s},y_{s}\right)\;\right\}$ consists of finding the parameters $m=\left(a_{0},a_{1}\right)$ so that the distance between the observed data $y^{\mathrm{obs}}=\left(\begin{array}{c} y_{1}\\ \vdots \\ y_{s} \end{array}\right)$ and the corresponding predictions $y^{\mathrm{pre}}\left(m\right)=\left(\begin{array}{c} y_{1}^{*}\left(m\right)\\ \vdots \\ y_{s}^{*}\left(m\right) \end{array}\right)$ is minimum according to the Euclidean distance in ${\mathbb{R}}^{s}$. The regression problem is equivalent to finding the least squares solution of the linear system of equations $Fm=y^{\mathrm{obs}}$, where the matrix $F=\left[1_{{\mathbb{R}}^{s}}\;\;x\right]$ depends on the abscissas of the data points $x=\left(\begin{array}{c} x_{1}\\ \vdots \\ x_{s} \end{array}\right).$

The uncertainty analysis of the least squares solution consists in sampling the family of equivalent models $m=\left(a_{0},a_{1}\right)$ that fit the observed data $y^{\mathrm{obs}}$ within the same error bounds:

(1) $$M_{tol}=\left\{m=\left(a_{0},a_{1}\right):\;\frac{y^{\mathrm{obs}}-y^{\mathrm{pre}}{\left(m\right)}_{2}}{y^{\mathrm{obs}}{}_{2}}<tol\right\}.$$

Fernández-Martínez et al. (2012, 2013) demonstrated that the topography of the data error cost function corresponds to a straight flat elongated valley if the inverse problem is linear, whereas in the nonlinear case, the cost function topography consists of one or more curvilinear valleys (or basins) of low misfits eventually connected by saddle points. In this simple linear regression problem, the equivalent models belong to an ellipse whose axes and orientations are related to the eigenvalues and eigenvectors of the matrix $F^{T}F$ [57,58,59]. 

This mathematical result might sound a little bit technical, but its importance lies in the fact that the uncertainty space of any decision problem has a deterministic structure. Furthermore, it can be shown that a simple way of sampling these equivalent model parameters in a linear regression problem consists of finding the least-squares solution of these different data bags. That is, solving a set of different regression problems with partial information, for example, with 75% of the observed data in fitting and the rest (25%) in validation (evaluating the predictive accuracy of this set of parameters). This procedure is named as 75/25 holdout (bootstrap) technique. 

**Figure 4:**
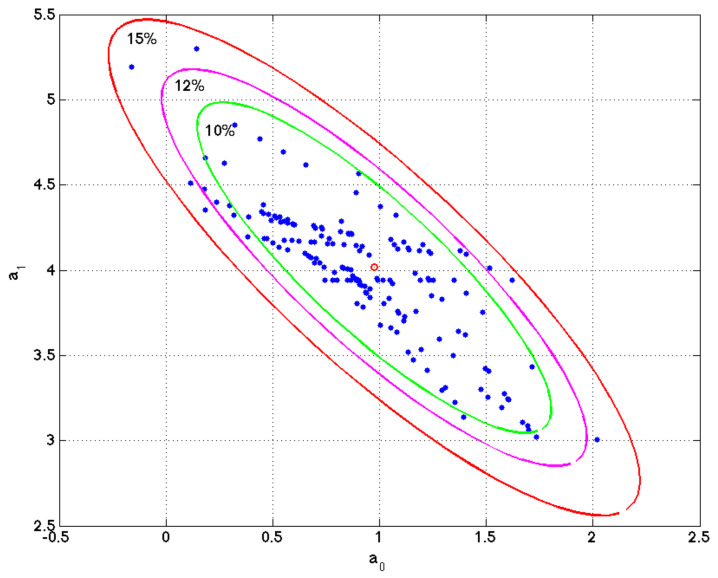
Linear regression model. Ellipse of uncertainty for a relative misfit of 15% and different sets of model parameters $\left(a_{0},\;a_{1}\right)$ found in the different bagging experiment. It can be observed that these models sample the region of uncertainty within the ellipse of 15% relative misfit. This example is very important to understand that the list of genes that equally explain a phenotype is not unique, and one simple method to sample these high discriminatory genetic networks is by performing random holdout, looking for the minimum-scale signatures that better explain the phenotype in each holdout, and finding the most-frequently sampled genes in these signatures, that are similar in phenotype prediction problems to the points (model parameters) located within the ellipse of this simple regression problem.

Figure 4 shows a numerical example of this procedure performed in simple linear regression problem. The figure shows the ellipse of model uncertainty for a relative misfit of 20%, and the different sets of parameters in the least squares fitting of different bagging experiments. It can be observed that these sets belong to the region of uncertainty and the sampling is denser along the axis of maximum uncertainty of this ellipse. 

Similarly, phenotype prediction problem can be viewed as a generalized regression between the discriminatory sets of genes that characterize the given phenotype and sets of sample classes that form the training data set [11]. As in the linear regression problem, one of the main obstacles in the analysis of genetic data is the absence of a conceptual model that relates the different genes/probes to the class prediction (phenotype). For this reason, a classifier $L^{*}\left(g\right)\;$ has to be constructed, as an algorithm that maps the set of genetic signatures g to the set of classes into which the phenotype is divided:

(2) $$L^{*}\left(g\right):g\in {\mathbb{R}}^{s}\to C=\left\{\frac{\mathrm{HC}}{\mathrm{LOAD}};\frac{\mathrm{HC}}{\mathrm{MCI}};\frac{\mathrm{HC}}{\mathrm{LOAD}+\mathrm{MCI}};\frac{\mathrm{LOAD}}{\mathrm{MCI}}\right\}.$$

In this paper we have performed four different comparisons: LOAD vs. HC, MCI vs. HC, LOAD vs. MCI, and LOAD + MCI vs. HC, to better understand the molecular mechanisms involved in LOAD and the main differences between LOAD and MCI.

Finding the discriminatory genetic signatures corresponding to $L^{*}\left(g\right)$ can be interpreted as a generalized regression problem of the observed sample class vector $\;c^{obs}$ with respect to the genetic signatures. For that purpose, the modeling is divided into 2 steps: learning and validation. The learning process consists of giving a subset of samples **T** (training data set) whose class vector $\;c^{obs}$ is known, and finding the subset of genetic signatures g, of minimum size that maximizes the learning accuracy (the percentage of samples with correctly predicted class):

(3) $$Acc\left(g\right)=100-L^{*}\left(g\right)-c^{\mathrm{obs}}{}_{1},\;\;$$

Here $L^{*}\left(g\right)-c^{\mathrm{obs}}{}_{1}$ stands for the prediction error (in percentage). In practice, the predictive accuracy of a genetic signature is established via cross-validation. Typically leave-one-out-cross validation (LOOCV) is used to use all the genetic samples that we have at disposal. The genetic signature with the highest accuracy and having the least number of discriminatory genes is named the smallest-scale signature. The validation consists in predicting the class of a new sample (whose class is unknown) using the genetic signatures that have been found during the learning process. The stability of the smallest-scale signature found at the learning step can be established via a holdout sampler procedure, where different samples for the training and validation set are randomly selected. 

It is important to remark that the phenotype prediction problems have always a high degree of underdeterminacy, since the number of monitored probes (genes) is much greater than the number of samples from human subjects enrolled in clinical trials. Therefore, the learning step involves several lists of genes with similar predictive accuracy. These genes might be involved in the genetic pathways associated with the disease. For a given classifier the smallest-scale signature is the one that has the least number of discriminatory genes with the highest predictive accuracy. This knowledge is very important for early diagnosis and treatment optimization. Due to noise in the genetic data and class assignment, some of these highly discriminatory signatures might be false, containing genes uninvolved in the genetic pathways [18]. 

To deal with this uncertainty problem we use bootstrapping methodology to sample the genes that are discriminatory in different holdouts and perform posterior analysis of these discriminatory networks to unravel the biological pathways that are involved in the disease development. This algorithm has been named holdout sampler [19] and has been used to sample the uncertainty space in various inverse problems [60,61]. It has been also successfully applied in phenotype prediction and drug design [62,63,64,65]. Besides, the predicted pathways are compared with those obtained using other sampling algorithms such as the Fisher’s ratio sampler [66] and Random Forest [67]. Random Forest (RF) are random decision trees for classification via ensemble learning. RF have been used for phenotype prediction and uncertainty analysis by Pang et al. [68]. These algorithms clearly outperform Bayesian Networks (BN) [69] that sample the posterior distribution of the genetic signatures related to the phenotype prediction, $P\left(g/c^{obs}\right)$, according to Bayes rule:

(4) $$P\left(g/c^{\mathrm{obs}}\right)\sim P\left(g\right).P\left(c^{\mathrm{obs}}/g\right),$$

Here $P\left(g\right)$ is the prior distribution of the genetic signatures, which is uniform in the set of discriminatory genes, or proportional to their discriminatory power, and $P\left(c^{obs}/g\right)$ is the likelihood of the genetic signature g, that depends on its predictive accuracy $\mathrm{Acc}\left(g\right)$ as follows:

(5) $$P\left(c^{\mathrm{obs}}/g\right)=ke^{Acc\left(g\right)}.$$

BNs have been used for the analysis of defective pathways by Jiang et al. [70] to discover gene interactions and by Su et al. [71] to analyze epigenetic modifications that affect the diseases. BNs have not used in this paper because they are very ineffective for pathways sampling and also very time consuming, due to optimization procedure of finding the optimum probability factorization. This procedure is inadequate for pathways sampling since the set of possible probabilistic factorizations of the uncertainty space is not unique. In fact, all the samplers follow implicitly equation [4] with different prior probabilities defined for different sets of discriminatory genes. Additionally, the likelihood $P\left(c^{\mathrm{obs}}/g\right)$, depends on the type of classifier and on the cost function that is used to define the predictive accuracy $\mathrm{Acc}\left(g\right)$.

**Figure 5:**
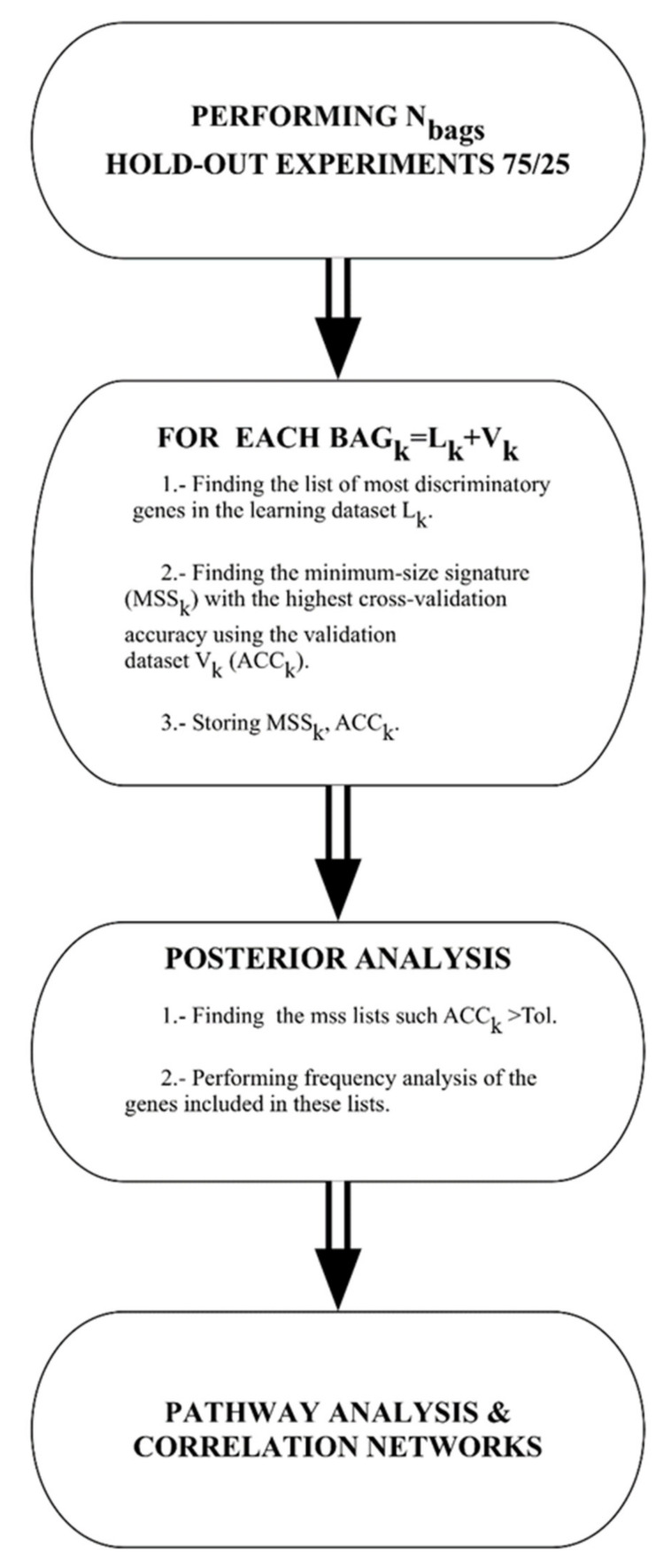
Flow chart of the machine learning methodology.

For all the algorithms used in this paper the flowchart is shown in Figure 5 and described as follows:

Data bagging: different random 75/25 data bags holdouts are generated, where 75% of the data is used for learning and 25% for validation. In the present study, we have used 1000 different bags. 

Gene selection: For each of these bags the genes are selected and the classifier is built. The set of genes that are used by different classifiers is first restricted to the most discriminatory ones to avoid the impact of genes that do not contribute mechanistically to the phenotype discrimination. The genes with Fisher’s ratio higher than a minimum cut-off (0.5 in this case) are selected. The accuracy is calculated on the validation set. This way of proceeding is based on the fact that variables with high discriminatory power serve to span the main features of the classification, while variables with lowest discriminatory ratios account for the details in the discrimination. This method determines the minimum amount of high-frequency details (helper genes) that are needed to optimally discriminate between classes promoting the header genes, which are those that explain the phenotype in a robust way. This methodology [11,18] has been successfully applied to the bioinformatics modeling of high-dimensional *Omics* data [72,73], and in the analysis of medical decision problems using hospital data [74,75]. 

Posterior analysis: once the data bagging simulation and analysis have been finished, the posterior analysis consists of finding all the minimum size signatures that have predictive accuracy of validation higher than a given tolerance. In this case, we have considered all the holdouts with predictive accuracy higher than 80%. Finally, we performed frequency analysis of these lists to find the most frequently sampled genes to establish the links with defective genetic pathways in Reactome Pathway Database.

## 5. Conclusions

In this paper, we present a novel algorithm to sample defective pathways involved in phenotype prediction problems that are highly underdetermined. The methodology consists of sampling different equivalent high-discriminatory genetic networks that are associated with the uncertainty space of the k-NN classifier that is used to separate the LOAD, MCI, and HC classes. To perform this task, the algorithm looks for the minimum scale signatures (header genes) corresponding to different 75/25 random holdouts. This methodology is related to bootstrapping techniques [76]. The biological pathways can be identified by performing posterior analysis, finding the most frequently sampled genes in these minimum scale signatures, and plugging these sets into ontological platforms. That way, the effect of helper genes whose presence might be due to noise or to the high degree of underdeterminacy of these experiments is damped. The proposed algorithm is very fast and simple to implement. However, a major problem is that predicted pathways may depend on the number of genes that are provided. Our experience recommends first using a sufficient number of representative genes. The second recommendation is to perform alternative analyses using variable numbers of genes to establish the cutoff frequency.

We show the application of this methodology to the analysis of the defective pathways in AD and MCI compared to healthy individuals. The results show common pathways for AD and MCI patients that are related to viral mRNA translation, influenza viral RNA transcription and replication, gene expression, mitochondrial translation, rRNA processing and metabolism. These pathways seem to be very consistent both for LOAD and MCI. The viral pathways have never been cited before among key pathogenic pathways involved in AD [9]. 

The predictive accuracies to discriminate LOAD and MCI from healthy controls were 84% and 81.5%, respectively. In addition, LOAD and MCI could not be clearly discriminated (74% accuracy). The most discriminatory genes of the LOAD-MCI discrimination are associated to regulation of apoptosis by protein ubiquitination and degradation and regulation of stress-activated MAP kinases by G protein signaling. This fact implies that MCI and LOAD can be diagnosed using similar pathways. We have also provided the list of genes that optimally discriminate LOAD and MCI from HC, which include the discriminatory genes that were found in individual discriminations of LOAD and MCI vs. HC. 

In conclusion, the retrospective analysis of this LOAD-MCI dataset using novel sampling approaches provides a new working hypothesis for the main genetic mechanisms involved in LOAD-MCI. Based on these findings we propose a set of repositioned drugs by using CMAP methodologies. The main drugs belong to the category of isoquinoline alkaloids, antitumor antibiotics, PI3K and autophagy inhibitors, antagonists of the muscarinic acetylcholine receptor, and histone deacetylase inhibitors. The mechanisms of action of these drugs are also detailed and correlated with specific pathways. 

We believe that the potential clinical relevance of these findings should be further investigated and confirmed with clinical studies of other independent cohorts using similar platforms to avoid possible biases. 

## Figures and Tables

**Table 1:** Late-onset Alzheimer’s Disease (LOAD) vs. healthy controls (HC). List of most discriminatory genes with a Fisher’s ratio higher than 0.8. All these genes are underexpressed in LOAD.

Gene	Mean-HC	Std-HC	Mean-AD	Std-AD	FC	FR (log)	Accuracy
*MRPL51*	703.3	183.20	472.4	121.55	0.57	1.29	78.71
*CETN2*	867.0	153.21	683.0	118.88	0.34	1.19	79.52
*LOC401206*	18,073.7	5002.52	12,083.0	4278.87	0.58	1.17	79.12
*RPL36AL*	7168.5	2178.39	4527.8	1711.00	0.66	1.16	78.71
*LOC646200*	3563.2	1854.66	1760.4	1074.05	1.02	1.09	79.12
*RPS25*	18,110.8	5359.11	11,732.2	4441.81	0.63	1.04	77.91
*RPA3*	450.8	111.78	332.4	80.43	0.44	0.99	77.11
*RPS27A*	17,344.8	3217.71	13,010.2	3989.02	0.41	0.96	77.11
*LOC653658*	1550.3	883.60	823.9	599.03	0.91	0.93	77.11
*LOC648000*	2445.1	1824.27	1119.2	1114.32	1.13	0.92	75.90
*LOC650276*	5630.2	3807.08	2744.5	2278.71	1.04	0.90	75.10
*MRPL33*	401.1	80.33	328.1	56.05	0.29	0.88	74.70
*RPL17*	3505.5	2669.20	1567.4	1607.24	1.16	0.87	74.30
*CALML4*	542.0	125.05	411.4	87.25	0.40	0.87	74.30
*RPL36AL*	2909.1	975.74	1789.1	743.33	0.70	0.86	74.70
*TOMM7*	3249.9	2034.70	1607.3	1223.54	1.02	0.85	74.30
*PSMC2*	815.7	229.18	610.1	179.02	0.42	0.84	78.31
*COX17*	1047.2	329.17	713.6	192.86	0.55	0.83	74.30
*SNRPB2*	869.3	239.44	649.1	161.49	0.42	0.83	77.51
*RPL6*	14,487.9	4110.82	11,020.4	3290.07	0.39	0.82	75.90
*LOC731365*	4091.6	1437.09	2900.3	1191.93	0.50	0.81	77.11
*ATP5J2*	1179.6	251.01	961.4	170.49	0.30	0.80	78.71
*LOC646483*	4230.1	1677.96	2885.7	1243.92	0.55	0.80	76.71

**Table 2:** LOAD vs. HC. Holdout sampler.

Gene	Mean H-C	Mean A-D	FC	FR	Frequency
*RPL36AL*	7168.47	4527.83	0.66	1.08	2.31
*MRPL51*	703.29	472.42	0.57	1.01	2.29
*CETN2*	867.00	683.01	0.34	1.05	2.27
*LOC401206*	18,073.70	12,082.96	0.58	1.12	2.26
*RPS27A*	17,344.77	13,010.21	0.41	1.07	2.18
*LOC646200*	3563.18	1760.36	1.02	0.53	1.99
*RPS25*	18,110.85	11,732.22	0.63	0.98	1.92
*LOC653658*	1550.32	823.92	0.91	0.61	1.83
*RPA3*	450.84	332.38	0.44	0.76	1.80
*RPL36AL*	2909.07	1789.05	0.70	0.88	1.54
*LOC648000*	2445.09	1119.19	1.13	0.42	1.44
*LOC731365*	4091.55	2900.26	0.50	0.65	1.44
*LOC650276*	5630.21	2744.46	1.04	0.56	1.42
*COX17*	1047.23	713.58	0.55	0.62	1.37
*CALML4*	542.04	411.37	0.40	0.69	1.37
*PSMC2*	815.75	610.09	0.42	0.68	1.33
*RPL17*	3505.48	1567.42	1.16	0.42	1.30
*MRPL33*	401.15	328.14	0.29	0.79	1.28
*ATP5J2*	1179.56	961.40	0.30	0.72	1.25
*SNRPB2*	869.31	649.12	0.42	0.63	1.24
*ATP5EP2*	11,802.46	7906.31	0.58	0.69	1.21
*MRPL33*	1461.80	1115.56	0.39	0.63	1.18
*ATP5O*	1523.69	952.53	0.68	0.53	1.16
*TOMM7*	3249.90	1607.26	1.02	0.41	1.10
*LOC646483*	4230.07	2885.69	0.55	0.59	1.08
*RPS17*	5544.05	3406.53	0.70	0.63	1.03
*METAP2*	595.99	449.62	0.41	0.56	1.03
*RPL6*	14,487.88	11,020.43	0.39	0.74	1.02
*GNL2*	388.71	320.97	0.28	0.71	1.01
*ING3*	376.85	282.53	0.42	0.56	1.00
*RPL6*	6640.20	4629.36	0.52	0.60	0.99
*PSMC6*	462.83	343.36	0.43	0.52	0.97
*FXR1*	297.54	249.31	0.26	0.66	0.93
*TINP1*	1437.50	1018.48	0.50	0.54	0.91
*NDUFA1*	3597.83	1604.43	1.17	0.32	0.84
*LOC648622*	2989.30	1499.56	1.00	0.48	0.79
*CCDC90B*	498.24	419.10	0.25	0.69	0.76
*DNAJA1*	1628.19	993.45	0.71	0.38	0.75
*RPL31*	1107.21	589.02	0.91	0.28	0.74
*SSBP1*	922.99	754.56	0.29	0.75	0.74
*LOC285900*	1831.82	1197.38	0.61	0.41	0.71

Most discriminatory genes sampled in different networks for the control vs. LOAD phenotype (sampling frequency higher than 0.7%). The sampling frequency is the number of times that a gene appears in the whole set of sampled genes. In this case a sampling frequency of 2.31% in a set of 43202 sampled genes in 1000 holdouts implies that this gene has been sampled 997 times. The sampling frequency could be also defined with respect to the number of holdouts and the first gene would have a sampling frequency of 99.7%. In any case this figure is a way of ranking the relative importance of each gene. We also provide the mean of the expression in each group, the fold change, the Fisher’s ratio, and the sampling frequency. All these genes are underexpressed in LOAD.

**Table 3:** LOAD vs. HC. Fisher’s sampler. Main genes found by the Fisher’s ratio sampler with a sampling frequency greater than 0.35.

Gene	Mean-HC	Mean-AD	FC	FR	Frequency
*HSP90AA1*	2538.09	1641.60	0.63	0.54	0.50
*PSMC6*	463.77	342.41	0.44	0.55	0.49
*LOC646483*	4228.56	2893.52	0.55	0.53	0.48
*RPL6*	6647.43	4635.21	0.52	0.55	0.48
*TMSB10*	11,572.19	9995.74	0.21	0.53	0.48
*ARPC3*	5021.07	3521.16	0.51	0.54	0.46
*RPL39*	5655.35	3956.08	0.52	0.54	0.46
*RAB37*	862.35	1065.21	−0.30	0.56	0.45
*SNRPB2*	867.06	650.20	0.42	0.55	0.44
*RPS20*	5026.68	3907.36	0.36	0.57	0.44
*NDUFA4*	1343.43	847.62	0.66	0.53	0.44
*GNL3*	272.90	234.36	0.22	0.55	0.44
*TCEAL4*	430.12	363.54	0.24	0.59	0.44
*ATP5F1*	1737.34	1318.29	0.40	0.56	0.43
*PCM1*	524.83	453.96	0.21	0.54	0.42
*PCMT1*	1268.60	1055.06	0.27	0.57	0.41
*RARS*	580.36	501.85	0.21	0.58	0.41
*BOLA3*	416.09	354.09	0.23	0.56	0.41
*KIAA0913*	882.61	1035.95	−0.23	0.56	0.40
*IGBP1*	501.94	404.70	0.31	0.56	0.40
*SCFD1*	638.10	539.69	0.24	0.58	0.40
*APBB3*	770.11	910.99	−0.24	0.58	0.39
*TRABD*	2274.77	2621.10	−0.20	0.58	0.38
*TROVE2*	312.54	351.15	−0.17	0.59	0.37
*NXF1*	1187.73	1373.17	−0.21	0.59	0.36
*TAX1BP1*	1297.46	961.12	0.43	0.60	0.35
*RPS27*	17,768.68	12,737.75	0.48	0.58	0.35
*SDCCAG10*	312.28	266.72	0.23	0.60	0.35
*C11ORF10*	3247.37	2775.15	0.23	0.57	0.35
*ACAT1*	392.11	314.89	0.32	0.57	0.35
*LOC729466*	3578.28	2644.66	0.44	0.59	0.35
*RPS17*	5544.94	3407.06	0.70	0.60	0.35

**Table 4:** LOAD vs. HC. Random Forest Sampler. Main genes found by the Random Forest sampler with a sampling frequency greater than 0.19.

Gene	Mean-HC	Mean-AD	FC	FR	Frequency
*LOC401206*	14.08	13.48	0.06	1.17	0.25
*MRPL51*	9.41	8.84	0.09	1.29	0.25
*CETN2*	9.74	9.40	0.05	1.19	0.24
*MRPL33*	10.47	10.09	0.05	0.76	0.24
*RPS27A*	14.06	13.60	0.05	0.96	0.24
*RPL36AL*	11.41	10.69	0.10	0.86	0.24
*RPL32*	13.43	13.12	0.03	0.70	0.23
*SNTB2*	9.35	9.63	−0.04	0.74	0.23
*RPL36AL*	12.74	12.05	0.08	1.16	0.23
*LOC388720*	14.19	13.74	0.05	0.67	0.23
*RPS25*	14.08	13.43	0.07	1.04	0.22
*BOLA2*	8.08	8.32	−0.04	0.71	0.21
*ATP6V1E1*	10.73	10.31	0.06	0.64	0.21
*PIGF*	8.20	8.02	0.03	0.55	0.21
*SSBP1*	9.82	9.53	0.04	0.77	0.21
*RPL6*	13.76	13.37	0.04	0.82	0.21
*NXF1*	10.19	10.41	−0.03	0.35	0.21
*PSMC2*	9.61	9.20	0.06	0.84	0.21
*SCFD1*	9.30	9.06	0.04	0.75	0.21
*MRPS17*	8.11	7.91	0.03	0.63	0.21
*COX17*	9.96	9.43	0.08	0.83	0.21
*ARPC3*	12.20	11.73	0.06	0.63	0.20
*RPS27*	13.96	13.48	0.05	0.44	0.20
*CWF19L2*	8.12	7.91	0.04	0.56	0.20
*AK2*	8.87	8.69	0.03	0.47	0.20
*NDUFA4*	10.11	9.54	0.08	0.61	0.20
*RARS*	9.15	8.95	0.03	0.59	0.20
*BOLA3*	8.68	8.44	0.04	0.64	0.20
*GNL3*	8.07	7.86	0.04	0.54	0.20
*CALML4*	9.05	8.66	0.06	0.87	0.20
*PCM1*	9.02	8.80	0.03	0.49	0.20
*CDC26*	9.53	9.25	0.04	0.68	0.20
*DNAJC7*	8.53	8.34	0.03	0.31	0.19
*SULT1A3*	8.37	8.63	−0.04	0.68	0.19
*SDCCAG10*	8.27	8.05	0.04	0.54	0.19
*MRFAP1L1*	9.82	9.47	0.05	0.55	0.19

**Table 5:** LOAD vs. Control. Pathways analysis obtained via different genetic samplers.

Sampler	LOAD vs. Healthy Control
Holdout sampler	Viral mRNA translation, Influenza viral RNA transcription and replication, Gene expression, Mitochondrial translation, rRNA processing in the nucleus and cytosol, Metabolism of proteins, Organelle biogenesis, HIV life cycle, Antigen, TCR signaling
Fisher’s sampler	Translation, Influenza life cycle, SRP-dependent co-translational protein targeting to membrane, Peptide chain elongation, Infectious disease, Influenza infection, Influenza viral RNA transcription and replication.
Random Forest	Selenoamino acid metabolism Viral mRNA translation Peptide chain elongation Selenocysteine synthesis Eukaryotic translation termination.

**Table 6:** Mild Cognitive Impairment (MCI) vs. HC. List of most discriminatory genes with Fisher’s ratio higher than 1.0. In this list only two genes are overexpressed in MCI (*RPS41Y* and *DENND1C*). Overexpressed genes are shown in bold.

Gene	Mean-HC	Std-HC	Mean-MCI	StdC-MCI	FC	FR	Accuracy
*TAX1BP1*	1300.3	479.32	833.5	326.66	0.64	1.47	69.02
***RPS4Y1***	1388.9	1508.67	1567.6	1416.45	−0.17	1.32	69.02
*LOC401206*	18,073.7	5002.52	12,518.9	3979.85	0.53	1.32	71.74
*RPL17*	3505.5	2669.20	1222.9	1204.77	1.52	1.24	67.93
*ATP5F1*	1737.2	487.80	1187.0	358.51	0.55	1.21	69.57
*SNX2*	828.8	220.12	625.0	130.12	0.41	1.20	70.65
*SUB1*	274.8	67.03	216.7	32.54	0.34	1.18	71.20
*LOC648622*	2989.3	2024.66	1182.5	870.25	1.34	1.14	69.57
***DENND1C***	415.9	93.32	504.0	82.49	−0.28	1.14	70.11
*LOC648000*	2445.1	1824.27	882.1	741.59	1.47	1.14	70.65
*VBP1*	442.5	127.57	329.6	78.39	0.42	1.14	69.02
*LOC650276*	5630.2	3807.08	2312.9	2015.90	1.28	1.12	69.57
*PSMC2*	815.7	229.18	584.5	161.75	0.48	1.12	69.02
*VBP1*	597.2	200.94	422.0	128.84	0.50	1.12	69.02
*LYPLAL1*	389.9	69.42	318.2	49.62	0.29	1.12	69.57
*HIGD1A*	472.3	134.84	354.7	85.62	0.41	1.11	70.11
*ATP6V1G1*	1137.9	494.51	653.5	253.77	0.80	1.10	69.57
*RPL31*	1107.2	801.24	440.5	334.26	1.33	1.10	68.48
*PNRC2*	873.2	273.86	621.0	211.62	0.49	1.09	69.02
*HSP90AA1*	2542.9	1091.30	1496.2	747.87	0.77	1.07	70.11
*RPS3A*	1492.7	948.63	752.6	625.26	0.99	1.07	70.65
*NDUFA4*	1342.2	772.90	645.2	291.73	1.06	1.07	70.65
*MRPL3*	537.4	197.46	366.9	120.14	0.55	1.07	71.20
*RPA3*	450.8	111.78	323.4	65.80	0.48	1.06	70.11
*MRPL33*	401.1	80.33	315.2	53.04	0.35	1.06	68.48
*LOC647340*	1228.3	530.94	754.3	349.22	0.70	1.06	68.48
*VAMP7*	544.7	134.08	420.3	89.05	0.37	1.05	67.93
*PSMC6*	462.8	179.02	313.5	116.15	0.56	1.04	67.93
*ANAPC13*	1056.1	248.28	773.8	178.28	0.45	1.03	67.93
*RPS25*	18,110.8	5359.11	12,048.6	3951.72	0.59	1.03	67.39
*VPS29*	987.2	340.14	668.5	209.15	0.56	1.03	67.93
*ACAT1*	392.9	104.10	299.3	65.65	0.39	1.03	67.93
*RPS3A*	1050.6	618.94	581.0	402.60	0.85	1.02	67.93
*CRBN*	468.9	127.79	352.1	92.43	0.41	1.02	69.57
*HINT1*	1849.4	1022.09	1046.0	741.48	0.82	1.00	71.74
*RPS27*	1562.3	1128.84	651.8	471.56	1.26	1.00	71.20

**Table 7:** MCI vs. HC. Holdout sampler.

Gene	Mean-HC	Mean-MCI	FC	FR	Frequency
*DENND1C*	415.91	503.99	−0.28	1.02	0.41
*LOC648622*	2989.30	1182.47	1.34	0.68	0.41
*LOC401206*	18,073.70	12,518.85	0.53	1.22	0.41
***SULT1A3***	337.68	413.27	−0.29	0.93	0.41
*ATP5F1*	1737.25	1186.98	0.55	1.04	0.41
*SNX2*	828.84	624.98	0.41	1.05	0.40
*HSP90AA1*	2542.86	1496.21	0.77	0.79	0.40
*LOC650276*	5630.21	2312.93	1.28	0.65	0.40
*LYPLAL1*	389.93	318.16	0.29	0.94	0.40
*TAX1BP1*	1300.26	833.54	0.64	1.29	0.40
*NDUFA4*	1342.20	645.23	1.06	0.73	0.40
*PSMC2*	815.75	584.52	0.48	0.88	0.40
*ANAPC13*	1056.07	773.77	0.45	0.96	0.40
*RPS3A*	1492.70	752.64	0.99	0.62	0.39
*TINP1*	1437.50	951.69	0.59	0.73	0.39
*VAMP7*	544.66	420.27	0.37	1.04	0.39
*RPS3A*	1050.62	581.04	0.85	0.61	0.39
*RPS27*	1562.31	651.80	1.26	0.45	0.39
*HIGD1A*	472.27	354.74	0.41	0.93	0.38
*MITD1*	429.65	343.49	0.32	0.90	0.38
*MRPL3*	537.40	366.90	0.55	0.77	0.38
*RPA3*	450.84	323.39	0.48	0.83	0.38
*IGBP1*	503.89	384.59	0.39	0.85	0.38
*LOC648000*	2445.09	882.14	1.47	0.53	0.38
*SLC35A1*	559.43	431.56	0.37	0.84	0.38
*PSMC6*	462.83	313.50	0.56	0.74	0.38
*RARS*	579.64	476.57	0.28	0.88	0.38
*UBE2E1*	932.67	641.56	0.54	0.73	0.38
*TMEM126B*	448.51	336.73	0.41	0.76	0.38

Most discriminatory genes sampled in different networks for the control vs. MCI phenotype (sampling frequency higher than 0.38%). We also provide the mean of the expression in each group, the fold change, the Fisher’s ratio, and the sampling frequency. Overexpressed genes are shown in bold.

**Table 8:** MCI vs. LOAD. List of most discriminatory genes with Fisher’s ratio higher than 0.25.

Gene	Mean-MCI	Std-MCI	Mean-AD	StdC-AD	FC	FR	Accuracy
*RPS4Y1*	1567.6	1416.45	1065.8	1351.05	0.56	1.45	62.22
*HLA-DRB1*	923.5	830.82	699.0	686.50	0.40	1.08	63.11
*JARID1D*	281.4	112.52	243.9	109.28	0.21	0.83	62.22
*HS.546019*	229.1	63.27	258.8	74.76	−0.18	0.56	62.22
*FYB*	2356.8	737.83	2861.7	823.29	−0.28	0.50	64.00
*XIST*	265.0	87.63	324.2	140.46	−0.29	0.44	61.78
*ZFX*	215.7	19.00	228.8	20.44	−0.09	0.37	64.44
*CCDC32*	226.1	17.09	236.4	20.15	−0.06	0.36	62.67
*SP3*	345.7	102.33	417.9	141.78	−0.27	0.36	64.89
*CREB5*	977.0	380.80	1243.8	457.64	−0.35	0.34	67.11
*STK17B*	357.1	71.26	402.5	97.13	−0.17	0.33	65.78
*LOC653855*	182.4	7.36	178.7	7.71	0.03	0.32	67.56
*ADAM10*	235.2	28.78	253.8	35.35	−0.11	0.32	66.67
*ACSL4*	370.0	107.89	451.5	158.92	−0.29	0.31	64.89
*RNF13*	476.8	113.40	558.3	151.25	−0.23	0.30	64.00
*FAM96A*	612.2	160.47	703.0	209.88	−0.20	0.29	64.89
*GNG10*	501.8	157.68	643.7	277.03	−0.36	0.29	64.44
*FBXO11*	872.8	212.75	990.0	224.71	−0.18	0.29	64.89
*HS.538581*	182.3	8.11	178.1	7.04	0.03	0.29	65.78
*WDR5*	325.9	26.74	312.1	29.54	0.06	0.28	66.22
*HS.537004*	331.4	62.88	371.1	81.60	−0.16	0.27	66.67
*CBFB*	389.1	83.57	433.8	89.55	−0.16	0.27	66.22
*CENTB2*	390.9	79.87	437.6	93.86	−0.16	0.27	65.33
*EML3*	1472.5	261.47	1333.6	277.31	0.14	0.27	64.89
*HS.460758*	169.5	6.21	174.0	7.99	−0.04	0.27	65.78
*HS.566890*	207.3	12.17	202.4	12.36	0.03	0.26	67.11
*ACD*	395.8	48.72	369.8	51.79	0.10	0.26	65.33
*DDX3X*	1225.7	390.46	1497.1	501.69	−0.29	0.26	66.22
*BAP1*	333.0	39.25	317.3	39.28	0.07	0.26	65.78
*AZIN1*	601.1	121.81	679.0	160.32	−0.18	0.26	64.89
*HS.130036*	284.1	55.50	318.8	67.07	−0.17	0.26	65.33
*DDX46*	284.7	34.04	300.7	35.77	−0.08	0.26	66.67
*RCBTB2*	204.5	16.37	211.9	16.37	−0.05	0.26	66.22
*AMD1*	544.7	142.10	613.1	166.94	−0.17	0.26	65.78

**Table 9:** MCI vs. LOAD. Most discriminatory genes sampled in different networks for the LOAD vs. MCI phenotype. Genes with sampling frequency higher than 0.5.

Gene	Mean-LOAD	Mean-MCI	FC	FR	Frequency
*HLA-DRB1*	699.02	923.49	−0.4	0.39	2.04
*FYB*	2861.74	2356.84	0.28	0.45	1.91
*HS.546019*	258.81	229.12	0.18	0.44	1.87
*CCDC32*	236.43	226.06	0.06	0.35	1.81
*SP3*	417.86	345.68	0.27	0.26	1.7
*RPS4Y1*	1065.75	1567.62	−0.56	0.72	1.68
*ZFX*	228.76	215.66	0.09	0.36	1.62
*XIST*	324.24	265	0.29	0.27	1.46
*CREB5*	1243.82	976.99	0.35	0.27	1.41
*ACSL4*	451.53	370.02	0.29	0.22	1.33
*LOC653855*	178.65	182.43	−0.03	0.31	1.25
*ADAM10*	253.83	235.2	0.11	0.3	1.19
*JARID1D*	243.87	281.42	−0.21	0.59	1.14
*RNF13*	558.32	476.84	0.23	0.26	1.14
*CBFB*	433.83	389.13	0.16	0.26	1.06
*STK17B*	402.48	357.12	0.17	0.29	1.04
*FBXO11*	990.02	872.76	0.18	0.27	1.02
*PRKY*	231.85	260.04	−0.17	0.16	0.94
*HS.130036*	318.83	284.09	0.17	0.22	0.94
*HS.537004*	371.06	331.41	0.16	0.25	0.83
*ACD*	369.76	395.81	−0.1	0.27	0.73
*GNG10*	643.73	501.82	0.36	0.17	0.71
*DDX3X*	1497.14	1225.72	0.29	0.24	0.71
*FAM96A*	703.02	612.23	0.2	0.2	0.69
*CENTB2*	437.57	390.9	0.16	0.27	0.69
*HS.538581*	178.06	182.28	−0.03	0.28	0.69
*WDR5*	312.12	325.87	−0.06	0.28	0.64
*SNORA25*	340.48	319.2	0.09	0.2	0.62
*BAP1*	317.32	333	−0.07	0.25	0.62
*HS.460758*	174.03	169.52	0.04	0.27	0.58
*B2M*	11,415.82	9954.21	0.2	0.19	0.56

**Table 10:** MCI+LOAD vs. HC.

Gene	Mean HC	Std HC	Mean MCI-AD	Std MCI-AD	FC	FR	Accuracy
*LOC401206*	18,073.7	5002.52	12,237.9	4171.3	0.56	1.22	73.86
*MRPL51*	703.3	183.2	480.2	121.31	0.55	1.19	76.9
*TAX1BP1*	1300.3	479.32	915.3	368.37	0.51	1.04	76.29
*RPS25*	18,110.8	5359.11	11,844.7	4267.77	0.61	1.04	76.6
*LOC650276*	5630.2	3807.08	2591	2194.12	1.12	1.02	75.68
*RPL36AL*	7168.5	2178.39	4716.9	1728.29	0.6	1.02	74.47
*RPA3*	450.8	111.78	329.2	75.53	0.45	1.01	75.99
*LOC646200*	3563.2	1854.66	1764.9	1008.41	1.01	1.01	75.08
*LOC648000*	2445.1	1824.27	1034.9	1002.56	1.24	1.00	75.08
*RPL17*	3505.5	2669.2	1444.9	1483.19	1.28	1.00	75.68
*MRPL33*	401.1	80.33	323.5	55.23	0.31	0.91	75.38
*LOC653658*	1550.3	883.6	801.8	531.34	0.95	0.91	75.99
*CETN2*	867	153.21	700.3	121.75	0.31	0.91	76.29
*CALML4*	542	125.05	410	83.48	0.4	0.89	76.6
*PSMC2*	815.7	229.18	601	173.15	0.44	0.89	76.9
*RPS27A*	17,344.8	3217.71	13,249.4	3883.87	0.39	0.88	74.77
*TOMM7*	3249.9	2034.7	1547.5	1174.84	1.07	0.87	74.77
*RPS17*	5544.1	2933.07	3241.9	2085.19	0.77	0.87	75.99
*SNX2*	828.8	220.12	651.9	168.48	0.35	0.87	75.68
*PSMC6*	462.8	179.02	332.7	132.18	0.48	0.87	76.6
*ATP5F1*	1737.2	487.8	1272.6	411.82	0.45	0.87	76.9
*TINP1*	1437.5	490.98	994.7	405.24	0.53	0.86	75.99
*SNRPB2*	869.3	239.44	641.7	151.97	0.44	0.85	77.2
*LYPLAL1*	389.9	69.42	330.2	57.48	0.24	0.85	75.38
*RPL31*	1107.2	801.24	536.2	491.17	1.05	0.84	75.38
*RPL36AL*	2909.1	975.74	1803.5	691.87	0.69	0.83	75.08
*RPL6*	14,487.9	4110.82	10,956.5	3335.45	0.4	0.83	75.68
*LOC648622*	2989.3	2024.66	1386.8	1104.76	1.11	0.83	75.38
*ATP5O*	1523.7	602.04	939	420.63	0.7	0.83	75.38
***SULT1A3***	337.7	65.75	406.1	64.14	−0.27	0.82	75.99
*GNL2*	388.7	72.09	319.2	46.16	0.28	0.82	75.68
*LOC646483*	4230.1	1677.96	2869.1	1207.02	0.56	0.82	75.99
*ACAT1*	392.9	104.1	309.3	70.1	0.35	0.82	75.68
*MRPL33*	1461.8	356.33	1113.6	260.92	0.39	0.82	75.68
*LOC388532*	902.7	532.22	499.6	301.65	0.85	0.81	75.68
*COX17*	1047.2	329.17	719.8	183.95	0.54	0.81	75.99
*SSBP1*	923	180.74	750.8	148.85	0.3	0.81	76.29

List of most discriminatory genes with Fisher’s ratio higher than 0.8. Only one gene (in bold face) is overexpressed in MCI + LOAD. This list contains several discriminatory genes found in each individual comparison (LOAD vs. HC and MCI vs. HC). Overexpressed genes are shown in bold.

**Table 11:** MCI + LOAD vs. HC. Most discriminatory genes sampled in different networks for the LOAD vs. MCI phenotype. Genes with sampling frequency higher than 0.36.

Gene	Mean HC	Mean MCI-AD	FC	FR	Freq.
*LOC653658*	1550.32	761.68	1.03	0.68	0.37
*LOC648622*	2989.3	1182.47	1.34	0.68	0.37
*PPP2R3C*	726.33	561.48	0.37	0.8	0.37
*LOC648000*	2445.09	882.14	1.47	0.53	0.37
*SULT1A3*	337.68	413.27	−0.29	0.93	0.37
*MITD1*	429.65	343.49	0.32	0.9	0.37
*LOC650276*	5630.21	2312.93	1.28	0.65	0.37
*VAMP7*	544.66	420.27	0.37	1.04	0.37
*SNX2*	828.84	624.98	0.41	1.05	0.37
*MRPL3*	537.4	366.9	0.55	0.77	0.37
*ATP5F1*	1737.25	1186.98	0.55	1.04	0.37
*EIF2A*	414.86	328.84	0.34	0.82	0.37
*NDUFA4*	1342.2	645.23	1.06	0.73	0.37
*DENND1C*	415.91	503.99	−0.28	1.02	0.37
*PSMC2*	815.75	584.52	0.48	0.88	0.37
*LOC401206*	18,073.7	12,518.85	0.53	1.22	0.37
*TAX1BP1*	1300.26	833.54	0.64	1.29	0.37
*ANAPC13*	1056.07	773.77	0.45	0.96	0.37
*SSBP1*	922.99	743.88	0.31	0.92	0.37
*RPS3A*	1492.7	752.64	0.99	0.62	0.37
*LYPLAL1*	389.93	318.16	0.29	0.94	0.37
*PNRC2*	873.24	620.99	0.49	0.93	0.37
*CRBN*	468.9	352.14	0.41	0.86	0.37
*HSP90AA1*	2542.86	1496.21	0.77	0.79	0.37
*PSMC6*	462.83	313.5	0.56	0.74	0.36
*RPL17*	3505.48	1222.94	1.52	0.56	0.36
*HIGD1A*	472.27	354.74	0.41	0.93	0.36
*DYNLT3*	260.74	216.35	0.27	0.74	0.36
*RPS27*	1562.31	651.8	1.26	0.45	0.36
*GPBP1*	610.75	470.14	0.38	0.79	0.36
*IGBP1*	503.89	384.59	0.39	0.85	0.36
*MRPL33*	401.15	315.19	0.35	0.96	0.36
*INPPL1*	780.71	958.41	−0.3	0.86	0.36
*SLC35A1*	559.43	431.56	0.37	0.84	0.36
*UBE2E1*	932.67	641.56	0.54	0.73	0.36

**Table 12:** Main results obtained for all the comparisons via the holdout sampler.

Item	LOAD vs. HC	MCI vs. HC	LOAD vs. MCI
Most Predictive Genetic Signature	*MRPL51, CETN2, LOC401206, RPA3, PSMC2, ATP5J2, LOC648622, SNTB2, LSM, EIF3E, DNAJA1, RPAP3, RPS17, ERCC5, LOC401397, RPS3A, SNRPD2, CCDC34, LOC440567, ATP5H, ANXA1.*	*TAX1BP1, LOC401206, RPL17, ATP5F1, LOC648622, VBP1, LOC650276, VBP1, ATP6V1G1, VAMP7, RPS25, RPS3A, LOC646483.*	*RPS4Y1, FAM96A, HS.460758, CLEC2A, SNORA25, SMCHD1, GIMAP2, MAP2K2*
Predictive Accuracy	84%	81.5%	74%
Pathways (High score matches)	Viral MRNA Translation, Influenza Viral RNA Transcription and Replication, Gene Expression, Mitochondrial translation, RRNA Processing in the nucleus and cytosol, Metabolism, Metabolism of proteins, Organelle Biogenesis, HIV Life Cycle, Antigen, TCR signaling.	Viral MRNA Translation, Gene Expression, Influenza Viral RNA Transcription and Replication, Metabolism of proteins, RRNA Processing in the Nucleus and cytosol, Ubiquitin-Proteasome Proteolysis, Antigen Processing, Cell cycle checkpoints, Metabolism, HIV Life Cycle, Mitotic Metaphase and Anaphase, CLEC7A (Dectin-1 signaling), Cellular Senescence, TCR signaling,...	Regulation of Activated PAK-2p34 By Proteasome Mediated Degradation, G-protein Signaling Regulation of P38 and JNK Signaling Mediated By G-proteins
Biological Processes (High score matches)	Translation, Nuclear-transcribed MRNA Catabolic Process, SRP-dependent Cotranslational Protein Targeting to Membrane, Proton Transport, Translation initiation, Viral Transcription, ATP synthesis, Mitochondrial Translation Termination and Elongation, ATP Biosynthetic Process, RRNA Processing,...	Translation, Nuclear-transcribed MRNA Catabolic Process, Translation initiation, termination and elongation, SRP-dependent Cotranslational Protein Targeting to Membrane, Viral Transcription, NIK/NF-KappaB Signaling, Regulation of MRNA Stability,	Positive Regulation of G1/S Transition of Mitotic Cell Cycle, Regulation of MRNA Stability.
Molecular Functions (High score matches)	Structural Constituent of Ribosome, Poly(A) RNA Binding, ATPase Activity, RNA binding, Protein Binding, ATP Synthase Activity.	Poly(A) RNA Binding, Protein Binding, RNA binding, Structural Constituent of Ribosome, ATP Activity.	Protein Binding, Translation Initiation Factor Binding.

**Table 13:** Main drugs identified by Robust Sampling of genetic pathways via Dr. Insights.

Drugs	Cell Line	Dose	Pathways
Cephaeline	MCF7	1 × 10^−7^	**Increased Expression** TRAIL signaling/Regulation of necroptotic cell death/Regulated Necrosis/RIP-regulated Necrosis/Interleukin-19,20,22 **Decreased Expression** Cellular Senescence/TP53 regulates transcription of genes involved in G1 cell cycle arrest/Cellular responses to stress
Tanespimycin	MCF7	1 × 10^−6^	**Increased Expression** Cellular response to heat stress/Regulation of HSF1 heat shock response/Unfolded protein response. **Decreased Expression** Apoptotic Execution/Processing of intronless pre-mRNAs/Signaling by TGF-beta receptor complex.
Wortmannin	MCF7-	1 × 10^−8^	**Increased Expression** Toxicity of botulinum toxin type D/IRS activation/Estrogen biosynthesis/Collagen biosynthesis/Growth hormone receptor signaling **Decreased Expression** RNA modification in the nucleus/Regulation of TP53 activity through methylation/Fatty acyl-CoA biosynthesis/Galactose catabolism
Biperiden	MCF7	1.15 × 10^−5^	**Increased Expression** Nuclear Pore Complex disassembly/Intraflagellar Transport/HIV life cycle **Decreased Expression** Secretin family receptors/Hemostasis/Adaptive immune system
Trichostatin A	PC3	1 × 10^−7^/1 × 10^−6^	**Increased Expression** Glutamate neurotransmitter release/Antigen activates B-cell receptor/CRMPs in Sema3A signaling **Decreased Expression** SMAD2/SMAD3:SMAD4 regulates transcription/G1 phase/Cyclin D associated events in G1/Transcriptional activation of mitochondrial biogenesis
LY-294002	MCF7	1 × 10^−7^/1 × 10^−5^	**Increased Expression** RNA polymerase I promoter opening/DNA methylation/Adaptive immune system/GPCR downstream signaling **Decreased Expression** Nucleotide-like (purinergic) receptors/P2Y receptors/Adaptive immune system

## Supplementary Materials

Supplementary materials can be found at https://www.mdpi.com/1422-0067/21/10/3594/s1.
