# Supplementary material for: Robust Sampling of Defective Pathways in Alzheimer’s Disease. Implications in Drug Repositioning
Source: Int J Mol Sci. 2020 May 19;21(10):3594. doi: 10.3390/ijms21103594 (PMC7279419; doi:10.3390/ijms21103594)
Supplement: Supplementary file 1 [file ijms-21-03594-s001.pdf]

Supplementary tables

Table S1. High score pathways in the LOAD vs. HC discrimination using the list of most frequently sampled genes with sampling frequency higher than 0.2 (93 identified genes).

Score	Path Name	# Total Genes	# Matched Genes
**46.96**	Viral mRNA translation	549	22
**27.89**	Influenza viral RNA transcription and replication	158	10
**27.34**	Respiratory electron transport, ATP synthesis	556	16
**24.50**	Gene expression	1842	27
**21.45**	Mitochondrial translation	95	7
**20.96**	rRNA processing in the nucleus and cytosol	203	9
**20.38**	Metabolism	2547	30
**17.92**	Metabolism of proteins	1630	22
**17.79**	Organelle biogenesis and maintenance	341	10
**16.54**	HIV life cycle	866	15
**15.27**	Antigen processing-Cross presentation	123	6
**15.20**	TCR signaling (REACTOME)	124	6

Table S2. High score pathways in the MCI vs. HC discrimination using the list of most frequently sampled genes with sampling frequency higher than 0.2 (161 identified genes).

Score	Path Name	# Total Genes	# Matched Genes
**49.83**	Viral mRNA translation	549	29
**33.20**	Gene expression	1842	43
**26.93**	Influenza viral RNA transcription and replication	158	12
**23.81**	Metabolism of proteins	1630	35
**23.03**	rRNA processing in the nucleus and cytosol	203	12
**20.30**	Ubiquitin-proteasome dependent proteolysis	122	9
**20.20**	Antigen processing-Cross presentation	123	9
**19.98**	Cell cycle checkpoints	204	11
**19.01**	HIV life cycle	866	22
**18.56**	Mitotic metaphase and anaphase	182	10
**18.28**	Mitotic G1-G1/S phases	145	9
**18.12**	CLEC7A (Dectin-1) signaling	147	9
**17.76**	Cellular senescence	454	15
**16.84**	TCR signaling (REACTOME)	124	8
**16.15**	Regulation of degradation of DeltaF508 CFTR	63	6
**15.77**	Activation of the mRNA	66	6
**15.52**	Signaling by hedgehog	141	8

Table S3. High score pathways in the MCI vs. LOAD discrimination using the list of most frequently sampled genes with sampling frequency higher than 0.5 (116 identified genes).

Score	Path Name	# Total Genes	# Matched Genes
**14.25**	Regulation of activated PAK-2p34 by proteasome mediated degradation	884	16
**12.28**	G-protein signaling regulation of P38 and JNK Signaling mediated by G-proteins	91	5
**10.99**	Plasma membrana estroben receptor signaling	26	3
**10.04**	Translation regulation by alpha-1 adrenergic receptors	74	4

Table S4. High score pathways in the LOAD+MCI vs. HC discrimination using the list of most frequently sampled genes with sampling frequency higher than 0.2 (154 genes).

Score	Path Name	# Total Genes	# Matched Genes
**51.42**	Viral mRNA translation	549	29
**31.19**	Influenza viral RNA transcription and replication	158	13
**29.78**	Gene expression	1842	40
**29.16**	Metabolism of proteins	1630	37
**22.92**	Metabolism	2547	44
**22.08**	HIV life cycle	866	23
**20.83**	Ubiquitin-proteasome dependent proteolysis	122	9
**20.74**	Antigen processing-Cross presentation	123	9
**20.68**	rRNA processing in the nucleus and cytosol	203	11
**19.32**	Respiratory electron transport, ATP synthesis	556	17
**19.14**	Mitotic metaphase and anaphase	182	10
**18.81**	Mitotic G1-G1/S phases	145	9
**18.65**	CLEC7A (Dectin-1) signaling	147	9
**17.70**	Cell cycle checkpoints	204	10
**17.31**	TCR signaling (REACTOME)	124	8
**17.16**	Regulation of PLK1 activity at G2/M transition	213	10
**16.53**	Chks in checkpoint regulation	224	10
**16.14**	Activation of the mRNA	66	6
**15.99**	Signaling by hedgehog	141	8
**14.68**	P53 signaling	117	7
**14.45**	RNA polymerase II transcription initiation	213	9
**14.38**	SMAD signaling network	121	7
**14.19**	Cellular senescence	454	13
**13.78**	Signaling By Wnt	340	11
